# Single Dose of SHR-1222, a Sclerostin Monoclonal Antibody, in Healthy Men and Postmenopausal Women With Low Bone Mass: A Randomized, Double-Blind, Placebo-Controlled, Dose-Escalation, Phase I Study

**DOI:** 10.3389/fphar.2021.770073

**Published:** 2021-10-20

**Authors:** Zhijie Dai, Pingfei Fang, Xiang Yan, Ronghua Zhu, Qiong Feng, Qiangyong Yan, Lingfeng Yang, Xiao Fan, Yuting Xie, Lihong Zhuang, Sheng Feng, Yantao Liu, Sheng Zhong, Zeyu Yang, Zhifeng Sheng, Zhiguang Zhou

**Affiliations:** ^1^ National Clinical Research Center for Metabolic Diseases, Key Laboratory of Diabetes Immunology, Ministry of Education, and Department of Metabolism and Endocrinology, The Second Xiangya Hospital of Central South University, Changsha, China; ^2^ Phase I Clinical Trial Center and Department of Pharmacy, The Second Xiangya Hospital of Central South University, Changsha, China; ^3^ Department of Clinical Research and Development, Jiangsu Hengrui Pharmaceuticals Co., Ltd., Shanghai, China; ^4^ National Clinical Research Center for Metabolic Diseases, Hunan Provincial Key Laboratory of Metabolic Bone Diseases, Department of Metabolism and Endocrinology, and Health Management Center, The Second Xiangya Hospital of Central South University, Changsha, China

**Keywords:** SHR-1222, low bone mass, sclerostin, bone resorption, bone formation

## Abstract

SHR-1222 is a humanized monoclonal antibody targeting sclerostin and has the potential to promote bone formation and reduce bone resorption. This study was aimed to assess the safety, tolerability, pharmacokinetics, pharmacodynamics, and immunogenicity of SHR-1222 in healthy men and postmenopausal women with low bone mass (BMD). It was a randomized, double-blind, placebo-controlled, dose-escalation, phase I study. Subjects received SHR-1222 at 50, 100, 200, 300, and 400 mg sequentially or matching placebo subcutaneously. Totally, 50 subjects with low BMD were enrolled and randomly assigned; 10 received placebo and 40 received SHR-1222 (50 mg, n = 4; 100, 200, 300, or 400 mg, n = 9). The most common adverse events that occurred at least 10% higher in subjects with SHR-1222 treatment than those with placebo were decreased blood calcium, blood urine present, increased blood cholesterol, electrocardiogram T wave abnormal, urinary tract infection, increased blood pressure diastolic, and positive bacterial test. All the above adverse events were mild in severity and well resolved except one of increased blood cholesterol in a subject lost to follow-up. The serum SHR-1222 concentration increased in a dose-dependent manner. Administration of SHR-1222 upregulated the bone-formation markers N-terminal propeptide of type 1 procollagen, osteocalcin, and bone-specific alkaline phosphatase, while downregulated the bone-resorption marker β-C-telopeptide. The BMD at the lumbar spine notably rose after a single dose of SHR-1222. The largest increase occurred in the 400 mg cohort (3.8, 6.7, and 6.1% on day 29, 57, and 85, respectively; compared with 1.4, 0.8, and 1.0% in the placebo group). Although 10.0% of subjects receiving SHR-1222 tested positive for anti–SHR-1222 antibodies, no obvious effects of antibody formation were found on pharmacokinetics. Overall, SHR-1222 was well tolerated at doses from 50 to 400 mg and is a promising new remedy for osteoporosis.

**Clinical Trial Registration:** http://www.clinicaltrials.gov, NCT03870100.

## Introduction

Osteoporosis is a skeletal metabolic disorder characterized by low bone density, reduced bone quality, and increased skeletal fragility and fractures (Armas and Recker, 2012). Many risk factors are associated with osteoporosis, including ageing, female gender, estrogen deficiency, alcohol, smoking, low body mass index, low dietary calcium intake, vitamin D deficiency, and insufficient exercise. Due to loss of protective effect of estrogen on bone, postmenopausal women are at a high risk of osteoporosis. It is estimated that the prevalence of osteoporosis at the spine or hip in China was 6.46 and 29.13% for men and women aged 50 years and older, respectively (Zeng et al., 2019). Approximately 50% of women and 20% of men with osteoporosis aged over 50 years will develop a fracture (Coughlan and Dockery, 2014).

Pharmacological treatments for osteoporosis are either anti-resorptive or anabolic drugs. Bisphosphonates, the most widely used anti-osteoporosis medication, inhibit bone resorption by inducing the apoptosis of osteoclasts actively engaged in the degradation of mineral on the bone surface and reduce the risk of fracture (Drake et al., 2008). However, patients should be noticed of gastrointestinal toxicities of oral administration, osteonecrosis of the mandible, and atypical femoral fractures caused by long-term use. Besides, the activity of osteoblasts is also blocked by bisphosphonates, which may subsequently lead to suppressed bone remodeling (Burr et al., 2003). Otherwise, anabolic drugs such as parathyroid hormone analogs and sclerostin inhibitors can promote bone formation and bone remodeling (Solling et al., 2019). Teriparatide, a typical parathyroid hormone analog, improves bone quality and bone mass by stimulating osteoblastic bone formation and reduces the risk of fracture in osteoporosis patients (Hodsman et al., 2005). However, the approved lifetime duration of treatment with teriparatide is limited to only 24 months because of the rat toxicology finding of osteosarcoma (Lindsay et al., 2016).

Sclerostin is not only a negative regulator of bone formation by upregulating the Wnt pathway, but also an enhancer of osteoclastogenesis by increasing the synthesis of receptor activators of NF-κB ligand (Poole et al., 2005; Paszty et al., 2010; Wijenayaka et al., 2011). Sclerostin inhibitors such as romosozumab have the prominent potential to enhance bone mass by promoting bone formation and reducing bone resorption (Padhi et al., 2011; Padhi et al., 2014). It has been demonstrated that romosozumab prevented new vertebral fracture more significantly than placebo or alendronate did in postmenopausal osteoporosis patients (Cosman et al., 2016; Saag et al., 2017). Currently, romosozumab is the only monoclonal antibody of sclerostin that has been approved by the US FDA and EU EMA since 2019 (EMA, 2019; FDA, 2019). Regrettably, romosozumab was found of possible cardiovascular disease risks, which may restrict its application (Saag et al., 2017). Moreover, romosozumab is not available in countries or regions outside the United States and the European Union. Developing alternatives to romosozumab is then an urgent need.

SHR-1222 is a newly generated humanized monoclonal antibody targeting sclerostin. Our preclinical studies showed that SHR-1222 could bind to human sclerostin with a high affinity (data on file, Jiangsu Hengrui Pharmaceuticals Co., Ltd.). This study was conducted to assess the safety, tolerability, pharmacokinetics, pharmacodynamics, and immunogenicity of SHR-1222 in healthy men and postmenopausal women with low bone mass.

## Methods

### Study Design

**FIGURE 1:**
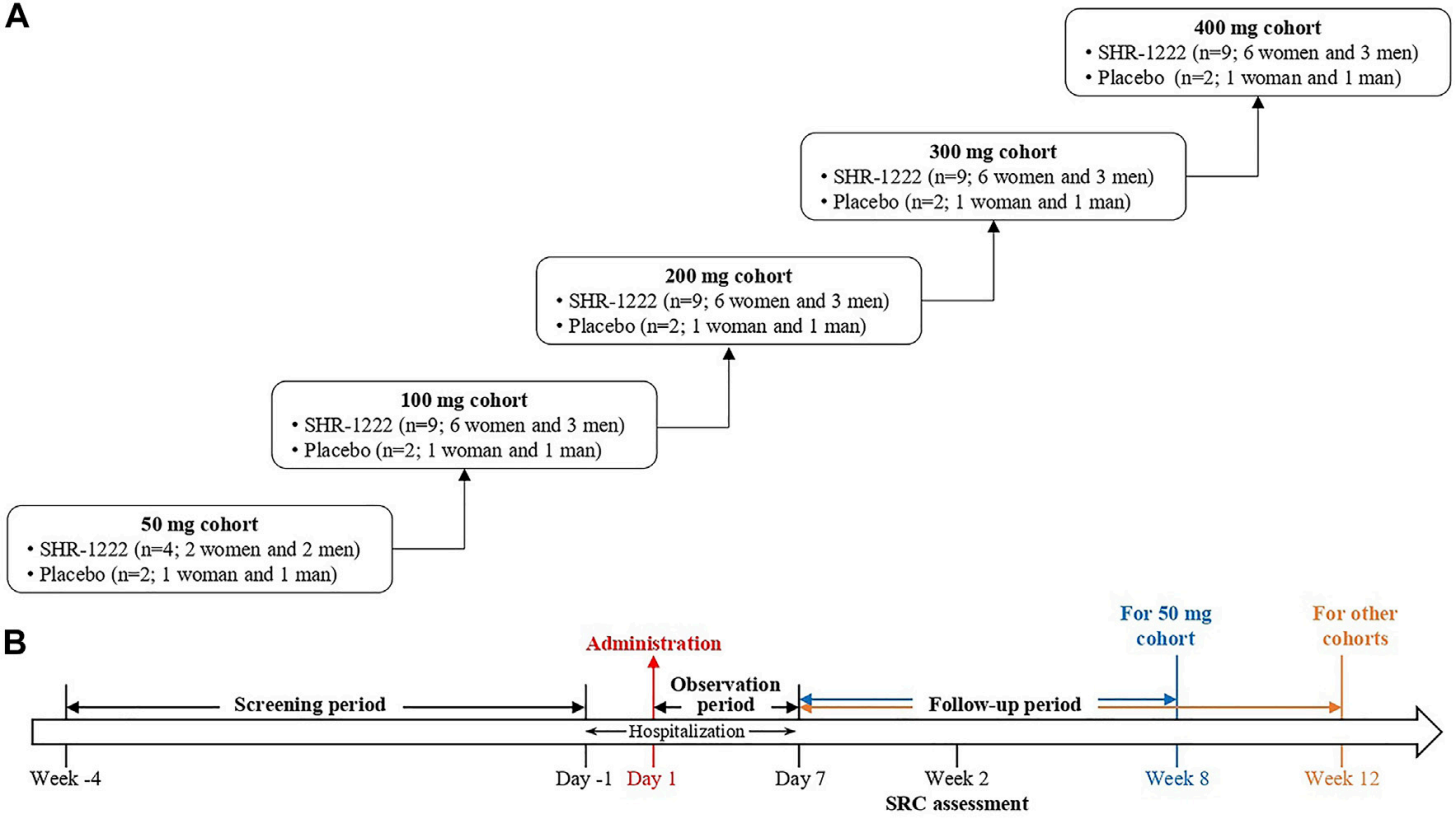
Study design. **(A)** Framework for the dose-escalation; **(B)** Dosing and assessment schema.

This was a randomized, double-blind, placebo-controlled, dose-escalation, phase I study of SHR-1222 in China (ClinicalTrials.gov Identifier: NCT03870100). Subjects received SHR-1222 at doses of 50, 100, 200, 300, and 400 mg sequentially or matching placebo subcutaneously (Figure 1). In the 50 mg cohort, 6 subjects were randomized, 4 to receive SHR-1222 (2 women and 2 men) and 2 to placebo (1 woman and 1 man). In each of the 100, 200, 300, and 400 mg cohorts, 11 subjects were randomized, 9 to receive SHR-1222 (6 women and 3 men) and 2 to placebo (1 woman and 1 man). The decision to proceed to the next dose cohort was made by the safety review committee composed of investigator and sponsor representatives after all subjects in the previous cohort had been monitored for at least 2 weeks since dosing.

The primary objectives of this study were to evaluate the safety and tolerability of a single subcutaneous injection of SHR-1222 and explore the safe dose range. The secondary objectives were to assess the pharmacokinetics, pharmacodynamics, and immunogenicity of SHR-1222.

The study protocol and all amendments were approved by the Ethics Committee of the Second Xiangya Hospital of Central South University and conducted in accordance with the Good Clinical Practice and Declaration of Helsinki. All subjects provided written informed consent before enrollment. The sponsor participated in study design and statistical analysis. All authors had access to the data, made contributions to the article writing or reviewing, and vouch for its accuracy and completeness.

### Study Population

Eligible subjects were healthy men or postmenopausal women between 45 and 59 years of age with low bone mineral density (BMD). Postmenopausal was defined as having bilateral oophorectomy 6 weeks before or having at least 12 months of spontaneous amenorrhea confirmed by a serum follicle-stimulating hormone concentration greater than 40 U/L. Low BMD was defined as a *T*-score between −2.5 and −1.0 (not including −2.5, −1.0) at the lumbar spine (L1–L4, one or more vertebra) or femoral neck, with modified WHO criteria (Kanis, 2002). Key exclusion criteria included any condition that would affect bone metabolism; prior estrogen/progesterone replacement therapy, calcitonin, parathyroid hormone and its analogs, doses of vitamin D greater than 1000 IU/day, glucocorticosteroids, anabolic steroids, or calcitriol and its analogs within the previous 6 months; prior bisphosphonates or fluoride for osteoporosis within the previous 12 months; a bone fracture within the previous 6 months; or a *T*-score of −2.5 or less at the lumbar spine (L1–L4) or femoral neck.

### Study Procedures

Laboratory examinations, vital signs, physical examinations, 12-lead electrocardiogram and echocardiography were performed for safety assessment. Adverse events were evaluated and coded according to MedDRA (Medical Dictionary for Regulatory Activities, version 22.0). Dual energy X-ray absorptiometry scans were performed for BMD values by the same Hologic Discovery Wi Bone Densitometer (Hologic, Bedford, MA, United States) at baseline, week 4 and 8 for the 50 mg cohort and at baseline, week 4, 8, and 12 for other cohorts. Instrument quality control was produced to ensure the coefficient of variation (CV) under 1% throughout the study. The least significant change of BMD was 2.8% according to the calculation based on the CV value (Hangartner, 2007).

Blood samples were collected on day 1 (pre-dose, 1 and 8 h post dose), 2, 3, 4, 5, 6 and 7 during the observation period, and from day 8 to week 12 (except for the 50 mg cohort, to week 8) during the follow-up period. Pharmacokinetic analyses were performed at the Shanghai InnoStar Bio-tech Co., Ltd., including time to maximum serum concentration (T_max_), maximum serum concentration (C_max_), area under the plasma concentration-time profile (AUC), half-life (t_½_), apparent clearance (CL/F) and apparent volume of distribution (V_d_/F), and mean residence time (MRT). PD biomarkers of N-terminal propeptide of type 1 procollagen (P1NP), osteocalcin (OST), bone-specific alkaline phosphatase (BSAP), and β-C-telopeptide (β-CTx) were analyzed at the Guangzhou KingMed Center using chemiluminescence methods (Roche Diagnostics Test Kits for P1NP, OST and β-CTx; Beckman Coulter Diagnostics Test Kits for BASP). Anti–SHR-1222 antibodies were determined at the Shanghai InnoStar Bio-tech Co., Ltd. using a validated Meso Scale Discovery electrochemiluminescence immunoassay.

### Statistical Analysis

All subjects who received placebo in different dose cohorts were combined into one placebo group. Subjects who had ever received SHR-1222 or placebo were included for safety analysis. Serum SHR-1222 concentration-time data were analyzed by noncompartmental methods. The PK analyses included all treated subjects for whom the PK parameters could be estimated. Percentage changes in bone metabolic markers and BMD from baseline were calculated. Data of safety, the serum SHR-1222 concentration, pharmacokinetic parameters, and percentage changes in PD biomarkers and BMD were grouped by the treatment and dose and summarized with descriptive statistics.

## Results

### Study Population

A total of 50 subjects with low BMD were enrolled and randomly assigned; 10 received placebo and 40 received SHR-1222 (50 mg, n = 4; 100, 200, 300, or 400 mg, n = 9). Of these subjects, 49 completed the study except one in the 100 mg SHR-1222 cohort due to loss to follow-up. Table 1 shows the demographics and baseline characteristics of the study population. There were 31 (62.0%) women and 19 (38.0%) men. The mean age of each cohort ranged from 50.0 to 52.4 years. The median *T*-score of each cohort ranged from −1.40 to −0.60 at the lumbar spine, −1.20–−0.60 at the femoral neck, and −0.90–0.10 at the total hip.

**TABLE 1:** Demographics and baseline characteristics.

	Placebo (N = 10)	SHR-1222					
		50 mg (N = 4)	100 mg (N = 9)	200 mg (N = 9)	300 mg (N = 9)	400 mg (N = 9)	All (N = 40)
Age, year, mean (SD)	50.8 (2.9)	50.0 (2.0)	51.9 (4.3)	52.4 (3.1)	51.7 (2.6)	50.3 (3.8)	51.4 (3.4)
Sex, n (%)							
Men	5 (50.0)	2 (50.0)	3 (33.3)	3 (33.3)	3 (33.3)	3 (33.3)	14 (35.0)
Women	5 (50.0)	2 (50.0)	6 (66.7)	6 (66.7)	6 (66.7)	6 (66.7)	26 (65.0)
BMI, kg/m^2^, mean (SD)	23.51 (1.98)	23.90 (2.38)	23.20 (1.44)	22.82 (1.47)	21.67 (2.18)	23.20 (2.61)	22.84 (2.04)
Lumbar spine L1-L4 BMD, median (min, max)	0.862 (0.735, 1.062)	0.918 (0.764, 0.961)	0.918 (0.820, 1.040)	0.879 (0.776, 0.993)	0.839 (0.752, 1.011)	0.826 (0.731, 1.041)	0.882 (0.731, 1.062)
Femoral neck BMD, median (min, max)	0.758 (0.617, 0.968)	0.753 (0.681, 0.864)	0.743 (0.589, 0.822)	0.692 (0.562, 0.834)	0.750 (0.635, 0.875)	0.738 (0.576, 0.812)	0.740 (0.562, 0.968)
Total hip BMD, median (min, max)	0.853 (0.756, 1.107)	0.858 (0.771, 1.013)	0.875 (0.689, 1.083)	0.804 (0.533, 0.970)	0.912 (0.744, 0.999)	0.858 (0.721, 0.987)	0.865 (0.533, 1.107)
Lumbar spine L1-L4 *T*-score, median (min, max)	−1.15 (−2.3, 0.7)	−0.65 (−1.9, −0.3)	−0.60 (−1.5,0.4)	−0.90 (−1.8, 0.0)	−1.30 (−2.0, 0.2)	−1.40 (−2.2, 0.4)	−0.90 (−2.3, 0.7)
Femoral neck *T*-score, median (min, max)	−0.75 (−2.0, 1.2)	−0.80 (−1.3, 0.1)	−1.10 (−2.1, −0.1)	−1.20 (−2.4, 0.1)	−0.60 (−1.9, 0.2)	−1.00 (−2.2, −0.1)	−0.95 (−2.4, 1.2)
Total hip *T*-score, median (min, max)	−0.45 (−1.7, 1.5)	−0.50 (−1.0, 0.6)	−0.10 (−1.7, 1.2)	−0.90 (−3.0, 0.7)	0.10 (−1.2, 0.6)	−0.30 (−1.5, 0.8)	−0.50 (−3.0, 1.5)

BMI, body−mass index; BMD, bone mineral density.

### Safety

SHR-1222 was well tolerated at all doses from 50 to 400 mg. All subjects experienced at least one adverse event during the study, but the majority were mild in severity. Only 7.5% (3/40) of subjects who received SHR-1222 had moderate or worse adverse events. No serious adverse events or adverse events leading to death occurred. No subjects withdrew from the study due to adverse events.

The most common adverse events that occurred at least 10% higher in subjects with SHR-1222 treatment than those with placebo were decreased blood calcium (37.5 versus 20.0%), blood urine present (27.5 versus 10.0%), increased blood cholesterol (22.5% versus 0), electrocardiogram T wave abnormal (15.0% versus 0), urinary tract infection (15.0% versus 0), increased blood pressure diastolic (10.0% versus 0), and positive bacterial test (10.0% versus 0) (Table 2). No dose-dependent trend was observed in the above adverse events except decreased blood calcium (25.0%, 0, 55.6, 44.4, and 55.6% in the 50, 100, 200, 300, and 400 mg cohort respectively). All the above adverse events were mild in severity and well resolved except one of increased blood cholesterol in the SHR-1222 100 mg cohort because the subject was lost to follow-up.

**TABLE 2:** Adverse events.

	Placebo (N = 10)	SHR−1222					
		50 mg (N = 4)	100 mg (N = 9)	200 mg (N = 9)	300 mg (N = 9)	400 mg (N = 9)	All (N = 40)
Any adverse event	10 (100.0)	4 (100.0)	9 (100.0)	9 (100.0)	9 (100.0)	9 (100.0)	40 (100.0)
Most common adverse events with a difference of at least 10% in incidence between SHR−1222 and placebo groups^a^							
Blood calcium decreased	2 (20.0)	1 (25.0)	0	5 (55.6)	4 (44.4)	5 (55.6)	15 (37.5)
Blood urine present	1 (10.0)	1 (25.0)	4 (44.4)	3 (33.3)	3 (33.3)	0	11 (27.5)
Blood cholesterol increased	0	2 (50.0)	3 (33.3)	1 (11.1)	2 (22.2)	1 (11.1)	9 (22.5)
Blood triglycerides increased^b^	4 (40.0)	3 (75.0)	2 (22.2)	2 (22.2)	0	1 (11.1)	8 (20.0)
Electrocardiogram T wave abnormal	0	0	1 (11.1)	0	4 (44.4)	1 (11.1)	6 (15.0)
Urinary tract infection	0	0	2 (22.2)	1 (11.1)	2 (22.2)	1 (11.1)	6 (15.0)
Blood phosphorus decreased^b^	4 (40.0)	0	0	3 (33.3)	0	1 (11.1)	4 (10.0)
Protein urine present^b^	2 (20.0)	0	1 (11.1)	3 (33.3)	0	0	4 (10.0)
Blood pressure diastolic increased	0	0	0	1 (11.1)	1 (11.1)	2 (22.2)	4 (10.0)
Bacterial test positive	0	0	1 (11.1)	2 (22.2)	1 (11.1)	0	4 (10.0)

a Adverse events that occurred in at least 2 subjects in either dose cohort of SHR−1222 group or placebo group and differed at least 10% in incidence between the SHR−1222 and placebo groups.

b Incidences were higher in subjects who received placebo compared with SHR−1222.

### Pharmacokinetics

**FIGURE 2:**
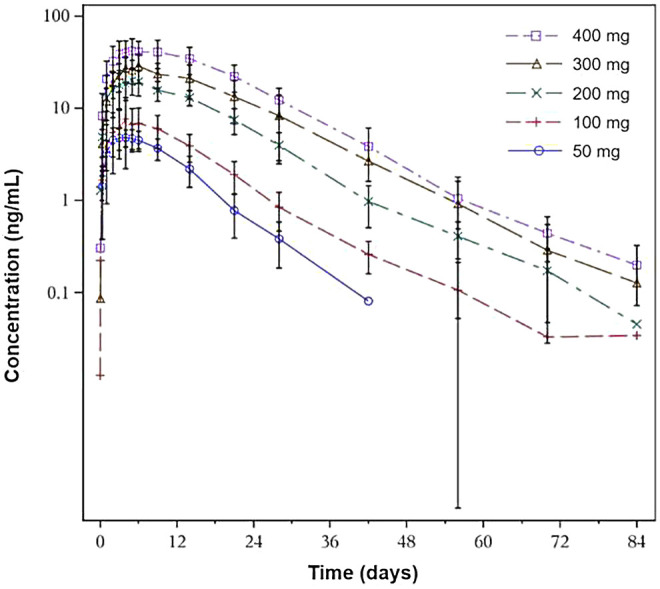
Mean serum concentration-time profiles of SHR-1222.

The pharmacokinetic parameters are summarized in Table 3. After a single dose of SHR-1222 ranging from 50 to 400 mg, serum concentrations in terms of C_max_, AUC_0-last_, and AUC_0-inf_ increased with dose. The serum SHR-1222 concentration increased in a dose-dependent manner (Figure 2; Table 3). Peak SHR-1222 serum concentrations occurred 3.5 days after administration in the 50 mg cohort and ranged from 5.0 to 6.0 days in the 100, 200, 300, and 400 mg cohorts. The mean t_1/2_ was 6.4 days in the 50 mg cohort and ranged from 9.0 to 10.4 days in other cohorts. The Geometric mean CL/F, V_d_/F and MRT ranged from 5.0 to 9.0 L/d, 6.2–11.5 L, and 10.5–16.6 days respectively.

**TABLE 3:** Pharmacokinetic parameters.

		SHR−1222				
		50 mg (N = 4)	100 mg (N = 8)	200 mg (N = 9)	300 mg (N = 9)	400 mg (N = 8)
T_max_, d	Median (Min, Max)	3.5 (2.0, 5.0)	6.0 (4.0, 8.1)	5.0 (1.0, 6.0)	6.0 (4.0, 13.1)	5.0 (4.0, 8.0)
C_max_, μg/mL	Mean (SD)	5.0 (1.1)	8.8 (5.2)	21.5 (7.0)	31.4 (9.5)	44.6 (14.4)
	GeoMean (%CV)	4.9 (22.5)	7.5 (59.3)	20.6 (32.5)	30.0 (30.1)	41.4 (32.3)
AUC_0−last_, d*μg/mL	Mean (SD)	64.9 (17.4)	117.0 (43.6)	368.0 (107.0)	605.0 (273.0)	947.0 (289.0)
	GeoMean (%CV)	62.9 (26.8)	108.0 (37.2)	357.0 (29.1)	558.0 (45.1)	882.0 (30.6)
AUC_0−inf,_ d*μg/mL	Mean (SD)	66.8 (17.4)	119.0 (43.5)	370.0 (107.0)	608.0 (274.0)	950.0 (290.0)
	GeoMean (%CV)	64.9 (26.0)	110.0 (36.5)	359.0 (28.8)	560.0 (45.0)	885.0 (30.5)
t_1/2_, d	Mean (SD)	6.4 (0.8)	9.0 (2.4)	10.4 (2.6)	9.7 (2.5)	9.6 (2.1)
	GeoMean (%CV)	6.4 (11.8)	8.7 (26.6)	10.1 (25.3)	9.7 (25.1)	9.4 (21.4)
CL/F, L/d	Mean (SD)	0.8 (0.3)	1.0 (0.6)	0.6 (0.1)	0.6 (0.2)	0.5 (0.4)
	GeoMean (%CV)	0.8 (32.1)	0.9 (59.9)	0.6 (21.0)	0.5 (36.9)	0.5 (68.8)
V_d_/F, L	Mean (SD)	7.2 (1.8)	13.9 (11.1)	8.6 (2.9)	8.3 (4.1)	7.3 (5.7)
	GeoMean (%CV)	7.1 (25.5)	11.5 (79.7)	8.2 (33.4)	7.5 (49.5)	6.2 (78.1)
MRT, d	Mean (SD)	10.6 (1.8)	13.9 (2.6)	15.2 (2.4)	16.2 (2.4)	16.7 (1.9)
	GeoMean (%CV)	10.5 (17.1)	13.7 (18.8)	15.0 (15.9)	16.0 (14.9)	16.6 (11.5)

AUC_0−last_, area under the concentration−time curve from zero to last time of quantifiable concentration; AUC_0−inf_, area under the concentration−time curve from zero to time infinity; CL/F, apparent clearance; V_d_/F, apparent volume of distribution; MRT, mean residence time.

### Bone Turnover Markers

**FIGURE 3:**
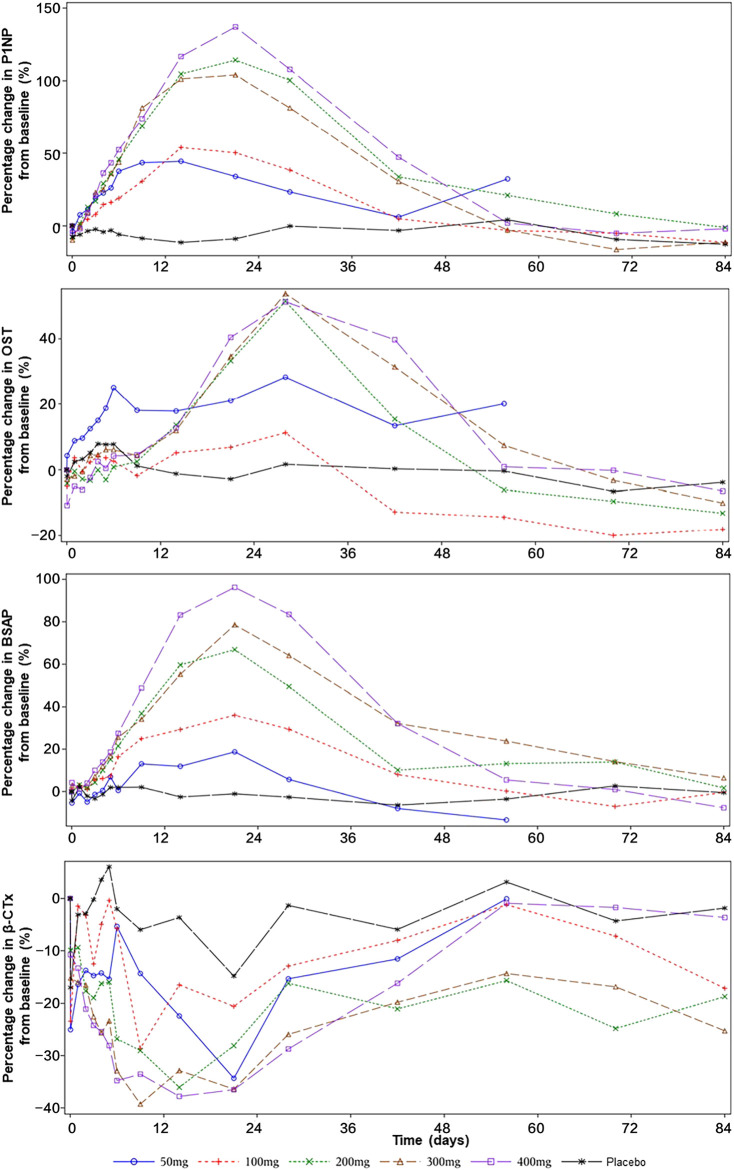
The effect of SHR-1222 on bone turnover markers. P1NP, N-terminal propeptide of type 1 procollagen; OST, osteocalcin; BSAP, bone-specific alkaline phosphatase; CTx, C-telopeptide.

Figure 3 shows the percentage changes in levels of the bone-formation markers P1NP, BSAP, and OST and bone-resorption marker β-CTx.

After a single dose of SHR-1222 from 50 to 400 mg, the serum levels of P1NP, OST, and BSAP increased from the baseline with the peak changes reached on day 15–22, day 29, and day 22 respectively. Also, dose-dependent increases were noted. The maximum changes of P1NP were 44.5, 54.0, 114.1, 103.9, and 137.0% in the 50, 100, 200, 300, and 400 mg SHR-1222 cohort respectively compared with 4.2% in the placebo group. Similarly, the maximum changes of BSAP were 18.7, 36.2, 66.9, 78.6, and 96.2% in the 50, 100, 200, 300, and 400 mg SHR-1222 cohort respectively compared with 2.7% in the placebo group. A little difference happened in the maximum changes of OST, which increased with the dose from 50 to 200 mg and reached a platform at doses of 200, 300 and 400 mg (28.2, 11.2, 51.2, 53.5, and 51.1% for 50, 100, 200, 300, and 400 mg, respectively; compared with 7.9% for placebo).

As for the bone-resorption marker β-CTx, its level decreased in all SHR-1222 cohorts after a single dose. The bottom levels were reached during day 10–22 (−34.3%, −28.6%, −36.1%, −39.3%, and −37.8% for 50, 100, 200, 300, and 400 mg, respectively; compared with −17.0% for placebo).

### BMD at the Lumbar Spine

**FIGURE 4:**
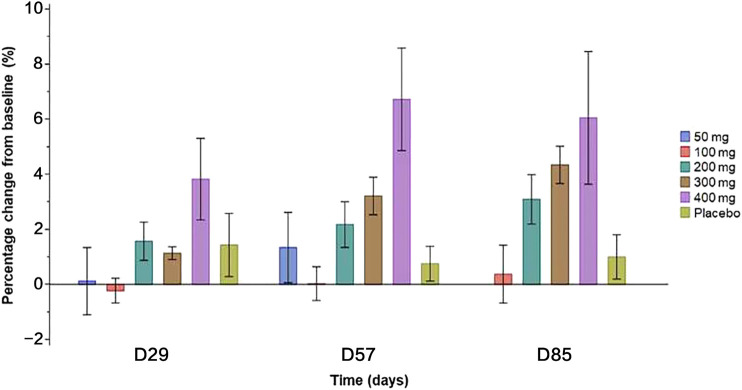
The effect of SHR-1222 on bone mineral density at lumbar spine L1-L4.

Compared with placebo, a single dose of SHR-1222 at 200, 300, and 400 mg caused increased BMD at the lumbar spine since day 29 (Figure 4), which appeared to be dose-dependent (Figure 4). The largest increase was observed in the 400 mg SHR-1222 cohort with the percentage change of 3.8, 6.7 and 6.1% on day 29, 57, and 85 respectively (compared with 1.4, 0.8 and 1.0% in the placebo group).

### Immunogenicity

Of the 40 subjects who received SHR-1222, 4 subjects (10.0%) tested positive for binding anti–SHR-1222 antibodies. However, no obvious effects of antibody formation were found on main pharmacokinetic parameters standardized by dose (AUC_0-last_/Dose, AUC_0-inf_/Dose, and C_max_/Dose).

## Discussion

This was a study assessing the safety and efficacy of SHR-1222 in healthy men and postmenopausal women with low BMD. Generally, SHR-1222 was safe and well-tolerated. Seven adverse events showed 10–22.5% higher incidences in subjects with SHR-1222 treatment than those with placebo. Among them, decreased blood calcium showed a dose-dependent trend. It was an expected adverse event for sclerostin inhibitors, which had been reported in the preclinical studies of SHR-1222 as well as the phase I study of romosozumab (Padhi et al., 2011). By direct blocking the interaction between sclerostin and low-density lipoprotein receptor-related proteins 5/6, sclerostin inhibitors could promote bone formation, inhibit bone resorption, and make blood calcium to be deposited in the bones, which eventually leads to a decrease in the serum blood calcium level (Winkler et al., 2003; Li et al., 2005; Semenov et al., 2005). Decreased blood calcium is a common adverse event in the treatment of osteoporosis, and vitamin D and calcium supplements are strongly recommended (Weaver et al., 2016; Paschalis et al., 2017; Cano et al., 2018). In phase III clinical trials of romosozumab for osteoporosis, daily calcium and vitamin D were given to all patients (Cosman et al., 2016; Saag et al., 2017). The incidence of hypocalcemia did not exceed 0.2% during the 2-years study period (Cosman et al., 2016; Saag et al., 2017). In our study, calcium and vitamin D were not supplied, and the incidence was 17.5% higher in subjects treated with SHR-1222 compared with placebo. This may indicate a powerful effect of SHR-1222 on calcium deposition in bone. Meanwhile, the adverse events of decreased calcium in this study were all mild in severity and transient in most of the subjects. No subject discontinued the study due to this event. In the following studies of SHR-1222, prophylaxis for decreased blood calcium should be considered by calcium and vitamin D supplementation.

Urinary disorders (blood urine present, bacterial test positive, and urinary tract infection) and cardiovascular disorders (electrocardiogram T wave abnormal and increased blood pressure diastolic) were unexpected adverse events for sclerostin inhibitors. However, in subjects aged between 45 and 59 indexes of urine system, blood pressure and electrocardiogram tend to be easily influenced by many factors. Slightly increased risks of cardiovascular events, heart attack, and stroke were observed in the efficacy trial of romosozumab (n = 4,093, Saag et al., 2017), which brought about a black box warning in romosozumab’s label (FDA, 2019). This kind of cardiovascular safety issue did not occur in this SHR-1222 study. Due to the small sample size, evidence is insufficient to say that SHR-1222 has disadvantages in the urinary tract or cardiovascular system. Further studies and more data are needed.

Compared with subjects receiving placebo, those with SHR-1222 showed a higher incidence of increased blood cholesterol but a lower incidence of increased blood triglycerides. The lower incidence of increased triglycerides could be explained as inhibition of sclerostin could increase fatty acid oxidation, reduce *de novo* fatty acid synthesis in adipocytes (Kim et al., 2017) and promote beige adipogenesis (Fulzele et al., 2017). The mechanism of the effect of SHR-1222 on cholesterol metabolism is unclear. Increased blood cholesterol found in this study was manageable: mild in severity; transient without obvious clinical meaningful impact; spontaneously resolved in all subjects except in one with the outcome unknown because of the loss to follow-up; and causing no withdrawal from the study. It is suggested that blood lipid levels should be monitored closely in the following studies.

After a single subcutaneous injection of SHR-1222, the serum level of SHR-1222 increased with the doses ranging from 50 to 400 mg in terms of C_max_, AUC_0-last_, and AUC_0-inf_. The increase ratio was greater than that of the dose. The T_max_ was 5.0–6.0 days and the half-life was 9.0–10.4 days at doses of 100–400 mg, similar to those parameters of romosozumab (5 and 12.8 days, respectively) (FDA, 2019).

SHR-1222 showed optimistic effects to promote bone formation, inhibit bone resorption, and increase bone density. The increase in BMD at the lumbar spine caused by SHR-1222 was similar to that by romosozumab at 3 mg/kg 3 months after a single dose (Padhi et al., 2011). Also, the maximum changes in P1NP, BSAP, and β-CTx following a single dose of SHR-1222 at 200 mg were comparable with those following a single dose of romosozumab at 3 mg/kg which is the approved recommended dosage for a 70 kg woman (Padhi et al., 2011).

SHR-1222 showed similar immunogenicity results compared with romosozumab. The drug-related anti-drug antibodies were detected in 10.0% of SHR-1222 and 11.0% of romosozumab treated subjects (Padhi et al., 2011). We did not find any obvious effect of antibody formation on pharmacokinetic parameters of SHR-1222. Due to the lack of data regarding SHR-1222 neutralizing antibodies and the small sample size in this study, further investigations are required.

In conclusion, a single subcutaneous injection of SHR-1222 was safe and well-tolerated within the doses ranging from 50 to 400 mg. Decreased blood calcium was a common adverse event of SHR-1222 in a dose-dependent manner, which may be prevented by supplementation of vitamin D and calcium. The pharmacodynamics analysis demonstrated the efficacy of SHR-1222 in BMD promotion, which supports the further clinical development of SHR-1222 as an attractive alternative treatment for osteoporosis. By the time of submission, another phase I study is in progress to assess the safety and efficacy of multiple doses of SHR-1222 in postmenopausal osteoporosis patients (ClinicalTrials.gov Identifier: NCT04435158).

## Data Availability

The datasets presented in this article are not readily available because of potential intellectual property. Requests to access the datasets should be directed to the corresponding authors.
